# The effect of 2 walking programs on aerobic fitness, body composition, and physical activity in sedentary office employees

**DOI:** 10.1371/journal.pone.0210447

**Published:** 2019-01-29

**Authors:** Mynor G. Rodriguez-Hernandez, Danielle W. Wadsworth

**Affiliations:** 1 Education Department, University of Costa Rica, San Ramón, Alajuela, Costa Rica; 2 School of Kinesiology, Auburn University, Auburn, Alabama, United States of America; Universitat de les Illes Balears, SPAIN

## Abstract

**Purpose:**

The present study examined changes in body composition, maximum oxygen uptake, and physical activity in sedentary office employees prescribed with two different walking programs during a 10-week intervention.

**Methods:**

68 sedentary employees were randomly assigned to one of three groups: multiple bouts of walking (n = 24 (5 male, 19 female) Age = 46±9, BMI = 30.5±5.78 kg/m^2^), continuous walking (n = 22 (6 male, 16 female) Age = 48±9, BMI = 30.6±6.2 kg/m^2^) and the control group (n = 22 (5 male, 17 female) Age = 42±10, BMI = 27.5±5.23 kg/m^2^). Dual-energy X-ray absorptiometry (iDXA) assessed body composition and a Bruce protocol treadmill test assessed aerobic fitness at baseline and week 11. At baseline, week 6 and week 11 a waist worn accelerometer measured physical activity and sedentary behavior. Physical activity was measured throughout the program with a wrist worn accelerometer.

**Results:**

The results from the mixed-design ANOVA show that fat mass (p < .000) and fat percentage (p < .000) decreased for all three groups as a main effect of time. Sedentary behavior did not change (p>0.05) for all three groups. Moderate intensity physical activity increased significantly from pre-test to week 6 (p<0.05), then decreased from week 6 to post-test (p<0.05), with no significant changes observed from pre-test to post-test (p>0.05) for all groups. No changes in VO_2_ were observed (p>0.05) for all groups.

**Conclusions:**

Continuous or intermittent walking activity produce similar benefits on body weight, fat mass and body fat percentage in sedentary employees. Meanwhile, intermittent walking allowed these sedentary employees to increase lean mass and fat free mass. Intermittent walking could provide at least similar benefits on body composition compared to a continuous walking program.

## Introduction

Sedentary behavior is considered a risk factor for developing non-communicative maladies such as cardiovascular disease, diabetes, osteoporosis, and other hypokinetic diseases [1–4]. Sedentary behavior is defined as low energy expenditure and the posture in which people remain for long periods of time either sitting or reclining [1]. Due to the relationship between chronic diseases and sedentary lifestyles, sedentary behavior is now considered a primary health detriment, linked to weight gain and excessive adiposity as well as other chronic negative outcomes [5, 6]. In addition, according to the Center for Disease Control and Prevention (CDC) [7] in 2013 only 20% of adults in the United States met the exercise recommendations for aerobic and muscle strengthening guidelines. Objective measures of physical activity show that less than 10% of the adult population in United States meet exercise recommendations [8]. This elevated rate of sedentary behavior coupled with low exercise participation rates has the potential for negative consequences on public health and quality of life [9].

Determinants of long bouts of inactivity have prompted studies to investigate interrupting sedentary time with physical activity intermittently. Intermittent physical activity can help people reduce the risk of premature diseases and early mortality [1, 10]. Specifically, reductions in waist circumference and body mass index, [11, 12], and other determinants of metabolic disease development [1, 2, 12–15] are associated with participation in intermittent exercise. Intermittent exercise bouts throughout the day appear to have similar or better physiological results compared to continuous forms of exercise as a single bout of exercise [11, 16]. In addition, it has been hypothesized that intermittent exercise bouts may lead to better adherence in terms of time management [2], and increase motivation due to the capability of performing short bouts of physical activity [17, 18]. Furthermore, short bouts of exercise may be easier for unfit people to perform, and incorporate it into their schedule [17], improving physical activity adherence.

Walking is a type of activity that can be used to disrupt sedentary behavior. Walking is related to many health benefits and quality of life while reducing the possibility of injuries or overstress [19, 20]. Walking is the most preferred physical activity [21], and a good alternative for people that are sedentary and/or never engaged in an exercise program before [22]. Multiple benefits are reported from walking interventions [23] including: changes in waist circumference [24], improvements in aerobic fitness [25], reduction in body fat and improvements in overall health [26]. For example, a 10 week physical activity intervention examined three different walking activities with matched volume and showed aerobic fitness improvements independently of the length of the bout [27] in another intervention, a two 45 minutes per week walking program prevented body fat increments in sedentary adults [28]. In addition, walking is a feasible activity for office employees, who spend a majority of their time sedentary and do not compensate for long hours of sedentary behavior by increasing physical activity outside work (19, 21). Previous studies that included walking or aerobic activity have mostly focused on continuous physical activity developed in one single bout (10 to 15 minutes) or long bouts [25, 27]. To our knowledge only a few studies have made comparisons between intermittent versus continuous physical activity and most of those programs have been completed in lab-based settings.

Although, some literature shows the effect of intermittent walking, there is limited knowledge on the effect of intermittent and continuous walking programs in ecological relevant environments on physiological variables, as well as, the effect of these walking programs on physical activity adherence within a program. Therefore, the purpose of this study to compare the effect of two different randomized exercise programs: intermittent walking and continuous walking in sedentary employees on physical activity behavior, VO_2_, and body composition, during a 10-week intervention.

## Materials and methods

### Participants, design, and study protocol

All procedures described herein were approved by the Auburn University Institutional Review Board for Human Subjects and conformed to the standards set by the latest revision of the Declaration of Helsinki. Prior to participation all subjects were asked to sign an informed consent and complete the Physical Activity Readiness Questionnaire (PAR-Q).

**Fig 1:**
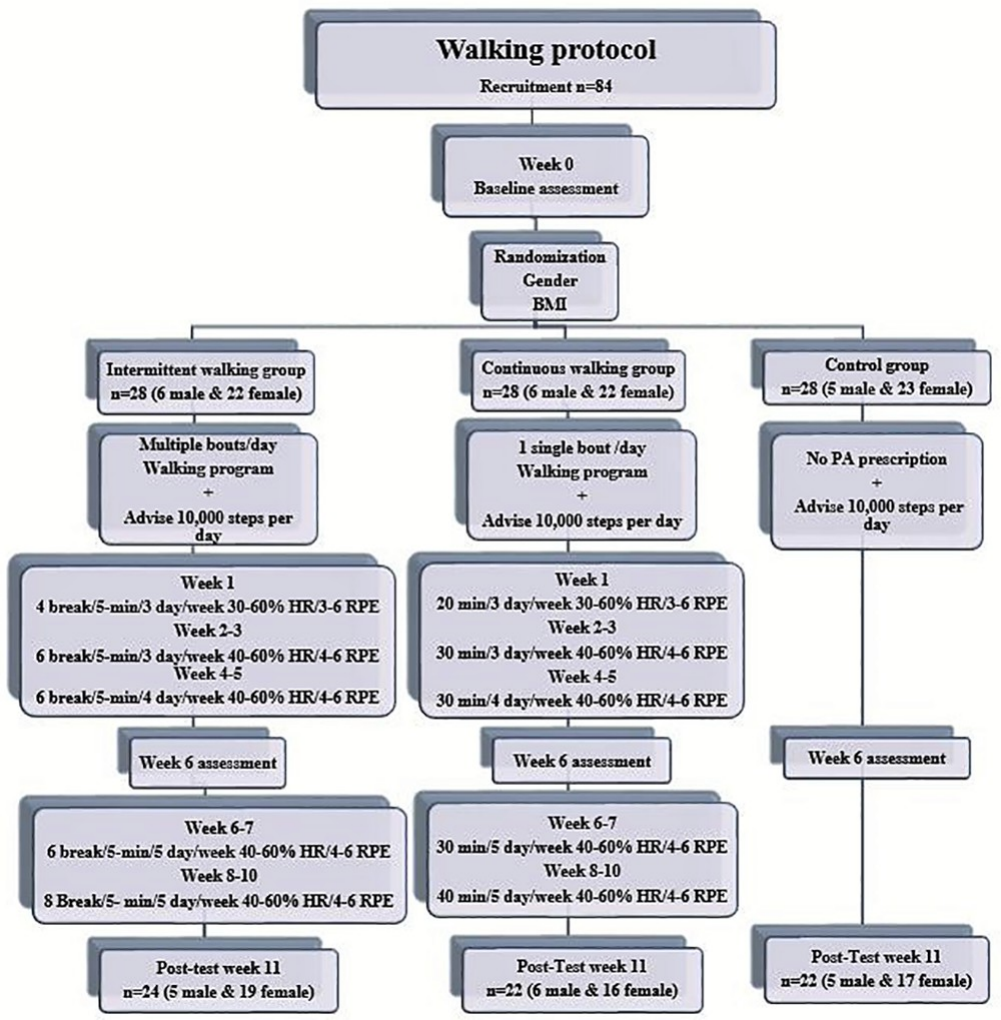
Study design.

This study involved 68 (females = 51 (75%), males = 17 (25%)) sedentary office employees who participated in a 10-week walking intervention. Subjects were randomly assigned to one of three groups consisting of two walking protocols; intermittent walking (n = 24 (5 male, 19 female) Age = 46±9, BMI = 30.5±5.78 kg/m^2^) and continuous walking (n = 22 (6 male, 16 female) Age = 48±9, BMI = 30.6±6.2 kg/m^2^), for both groups time and intensity were matched. A third group served as the control group (n = 22 (5 male, 17 female) Age = 42±10, BMI = 27.5±5.23 kg/m^2^) and were not prescribed with a physical activity program. The sample size was determined following Cohen’s recommendations [29]. Then, for a conservative calculation a_1_ = .05, r = .30, and power = .80 the desire sample size was 68. The initial sample was established considering the attrition rate range for interventional studies, which was estimated by Linke, Gallo and Norman about 18–34% [30] or the reported general attrition for interventional studies of 25–50% [31] Randomization was designed to equate males and females and body mass index (BMI) status among the three groups. The exercise prescription for the two walking groups consisted of an incremental walking program that increased frequency and duration across the ten weeks (Fig 1). All walking exercises were completed independently by the participants. To control walking intensity both intervention groups were trained on how to perform physical activity using the Rate of Perceived Exertion (RPE) scale to calculate the intensity of the activity. During baseline testing upon the return of the waist worn accelerometer, participants completed an incremental treadmill protocol until the participant reached a moderate pace based on heart rate measures. This was the pace prescribed to the participant for the walking prescription.

Baseline and post assessments (week 11) included height and weight assessed using a stadiometer (SECA Model 769, Seca gmbh & Co.kg., Hamburg, Germany), body composition via dual-energy X-ray absorptiometry (iDXA) (GE Healthcare Lunar, Madison, WI), aerobic fitness via a submaximal oxygen consumption (VO_2_) test, and daily physical activity via a waist worn accelerometer (ActiGraph GT3X; ActiGraph Corp., Pensacola, FL). Waist worn accelerometers were also worn for 7 days at week 6. In addition, weekly steps were measured with a Movband (DHS Group, Houston, TX) which is a wrist worn accelerometer). Fig 1 explains the protocol design.

### Assessments

#### Aerobic fitness

Aerobic fitness was measured with a submaximal Bruce Protocol (volitional fatigue). This protocol reports a standard error of estimates (SEEs) range from ±2.7 to ±4.7 mL*Kg^-1^*min^-1^ [32]. Aerobic fitness was estimated by asking the participant to walk- jog, and/or run on a treadmill for three-minute stages, beginning at 10% incline and 1.7 miles per hour (MPH). At the end of each stage incline and MPH was increased [33]. Once the participant reached maximum fatigue tolerance, the test was stopped and VO_2_ was estimated by using a standardized and validated formula [VO_2_ (mL*kg^-1^*min^-1^) = 6.7–2.82(2) + .056(time in seconds)]; [Women VO_2_ (mL*kg^-1^*min^-1^) = 1.06 + .056(time in seconds)]; [Men VO_2_ (mL*kg^-1^*min^-1^) = 3.88+ .056(time in seconds)] [33].

Although, submaximal exercise testing is not as precise, it provides a general idea on a person’s aerobic fitness, reduces cost, reduces risk of negative events, needs less time and effort on the part of the subject, and assumptions related to submaximal test are easily met. In addition, this cohort was sedentary upon initiation of the test. According to ACSM [34] when a repeated submaximal GXTs are applied over a period of weeks or months and with a HR response decreasing over time with a fixed workload, it is likely that the aerobic fitness of that person improved.

#### Body composition

Body composition was assessed by iDXA, which provides data related to body composition in terms of BMI, body fat, lean mass and bone mineral density. We have previously reported [35] that test-re-test reliability of the iDXA on 10 participants produced intra-class correlation coefficients of 0.998 for total body fat mass [mean difference between tests (mean ± standard error) = 0.40 ± 0.05 kg] and 0.998 for total body lean mass [mean difference between tests (mean ± standard error) = 0.29 ± 0.13 kg].

#### Physical activity measures

Physical activity was measured with a waist worn and wrist worn accelerometer. To measure physical activity and sedentary behavior an Actigraph accelerometer GT3X (ActiGraph GT3X; ActiGraph Corp., Pensacola, FL) was attached on the right hip of each participant to assess changes regarding sedentary, light, moderate, and vigorous physical activity at baseline, week 6 and week 11. The device is a small trial-axial device weighing 27g and measuring 3.8 cm x 3.7 cm. x 1.8 cm. The GT3X records accelerations ranging from 0.05 to 2 g at a rate of 30 Hz in three different axes: vertical, antero-posterior, and medio-lateral [36]. Based on previous studies and best practice guidelines [37, 38], an epoch length of 1-minute was chosen as the standard for the current study with a sampling rate of 30 Hz. Additional criteria for analysis include a minimum of 10 hours daily wear time and 3–5 days of monitoring. There is relative consensus of a minimum of 10 hours per day of wear time needed for sampling wake-time behavior with 3–5 days of monitoring required to achieve 80% reliability for total and moderate-to-vigorous intensity physical activity [39–41]. Participants were asked to wear the device during all waking hours with the exception of showering and engaging in other water-based activities (i.e. swimming). Non-wear time was identified by participants completing a daily log of wear time and the Choi et al. algorithm [42]. Non-wear time was removed from the analysis. The remaining data were analyzed as described below in the statistical analysis. Previously validated cut points were used to classify accelerometer data as sedentary (<100 counts/minute), moderate (<5,999 counts/minute) and vigorous (>5,999) [8]. Light activity was defined as 500–2019 counts per minute [43].

A second device was given to the all three groups to track daily activity during the entire intervention. A Movband (Movband; DHS Group, Houston, TX.) is a wrist-worn activity monitor that measures daily physical activity and reports that activity as “moves”. Approximately 12,000 moves are equal to 10,000 steps. The Movband is attached to the wrist with a watch band and records steps and moves per day. Participants were asked to wear the device throughout the intervention and remove it only when swimming below 25 meters. Unlike the actigraph, wear time cannot be teased out from the data, however, participants reported they used the device as it was required and Movbands were synced each week. Reliability for the Movband on a treadmill has been reported as r = 0.92, p<0.02 [44], and for free living PA as r = 0.974 [45]. Participants used a username and password to log in, sync, charge and download recorded information each week via cloud-based software. Each group could see daily physical activity for themselves and for members of their group, but not members of the two other groups. Participants in the two experimental groups were given a Movband and the walking prescription according to the intervention group. Participants in the control group were given the Movband but did not have access to a walking program. Movband data was monitored for the duration of the study and moves were recorded and presented for comparison purposes as weekly means per group.

### Statistical analysis

All analyses were performed with SPSS 23.0. To answer the research questions, a mixed design ANOVA approach was performed to examine the main effect over time and the main effect of time and group interaction. Between factors examined differences between the three groups, whereas, within factors assessed change over time. When a significant main effect (i.e. p<0.05) was observed, a Post-Hoc test was performed using Bonferroni correction for multiple comparisons. To analyze the effect of 10-week intervention on sedentary office workers, three groups were randomly established: intermittent walking, continuous walking, and a control group to analyze the following variables: aerobic fitness, body composition (lean mass, fat mass, visceral fat, android fat and gynoid fat), percentage of time spent in sedentary behavior, light, moderate, and vigorous intensity physical activity and steps average per week measured by the Movband.

## Results

From the initial sample, sixty-eight sedentary office employees completed the intervention. At the onset of the study, groups did not differ by BMI p = 0.272. The adherence and attrition rates were 80.95% and 19.05% respectively, which is considered positive rates according to the general acceptable rates [30, 31]. The sixty-eight participants who completed the study synced the Movband device each week intervention and completed all measurements.

### Aerobic fitness results

**Fig 2:**
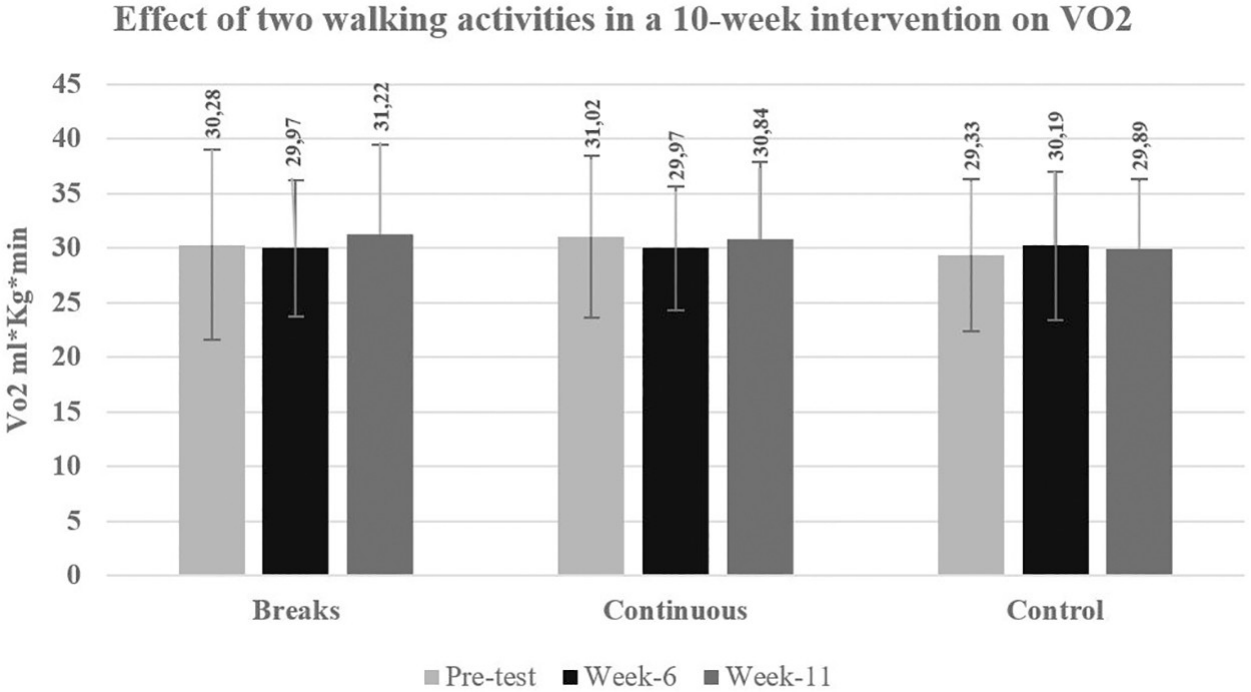
Oxygen uptake measured by Bruce protocol.

The results from the mixed-design ANOVA show no significant changes for VO_2_ measured by the Bruce Protocol (Fig 2). There was no main effect of group *F*(2,65) = .091, p = 0.913, or of time *F*(1.62,105.07) = .997, *p* = 0.358) or time by group interaction (*F*(3.23,105.07) = 1.060, *p* = 0.373).

### Body composition results

The iDXA results show significant main effect of time changes for the three groups on total weight, fat mass, lean mass, fat percentage, android fat, and gynoid fat. Only lean mass showed main effect of time by group interaction changes *F*(2,64) = 3.899, p = 0.025. Post-Hoc showed significant improvements in the intermittent (p = .000) and control group (p = 0.020) as shown in Table 1.

**Table 1:** Body composition main results obtained by iDXA scan.

		PRE-TEST	Week-11	Main effect of group			Main effect of time			Time by group interaction			Pre-test–Week-11
				F	p	n^2^	F	P	n^2^	F	P	n^2^	p
**Overall Mixed ANOVA**	Total weight Kg.			1.489	.233		3.416	.069		1.568	.216		
	Fat mass Kg.			1.022	.365		24.829	.000*	.276	1.726	.186		
	Lean mass Kg.			1.283	.284		12.881	.001*	.165	3.899	.025*	.107	
	Visceral fat Kg.			0.141	.869		1.960	.166		0.590	.558		
	Fat %			1.633	.203		36.179	.000*	.358	1.633	.203		
	Android fat %			0.485	.618		21.193	.000*	.246	1.693	.198		
	Gynoid fat %			0.850	.432		10.601	.002*	.140	1.013	.369		
**Breaks**	Total weight Kg.	82.99±18.8	82.51±19.16										
	Fat mass Kg.	34.63±10.46	33.48±10.61										
	Lean mass Kg.	45.7±10.76	46.4±11.19										.000**
	Visceral fat Kg.	1.23±0.73	1.17±0.71										
	Fat %	42.54±6.61	41.33±6.93										
	Android fat %	48.76±8.82	46.76±9.82										
	Gynoid fat %	45.07±7.38	43.88±7.59										
**Continuous**	Total weight Kg.	87.70±21.96	86.62±21.01										
	Fat mass Kg.	35.26±13.06	34.21±12.64										
	Lean mass Kg.	49.69±12.6	49.66±12.11										
	Visceral fat Kg.	1.27±0.97	1.19±0.96										
	Fat %	40.78±8.96	40.07±8.96										
	Android fat %	45.64±12.91	44.58±12.88										
	Gynoid fat %	44.00±9.64	43.56±9.94										
**Control**	Total weight Kg.	77.40±15.09	77.53±15.42										
	Fat mass Kg.	30.33±11.41	29.92±11.46										
	Lean mass Kg.	44.52±7.48	45.07±7.79										.020**
	Visceral fat Kg.	1.09±1.06	1.09±1.03										
	Fat %	39.49±9.57	38.84±9.81										
	Android fat %	44.85±14.07	44.02±14.26										
	Gynoid fat %	41.52±9.22	40.91±9.46										

Mixed ANOVA results are presented at the top of the table, degrees of freedom are: main effect of group (2,65), main effect of time (1,65), and time by group interaction (2,65).

* Significantly different (p < .05)

** unique time by group interaction, significantly different (p<0.05)

### Accelerometer and Movband results

#### Accelerometer

**Fig 3:**
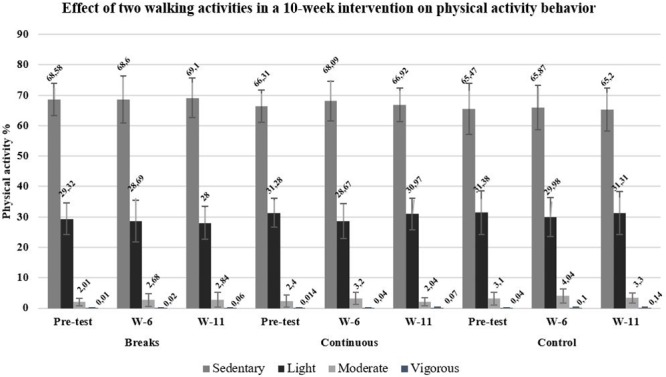
Physical activity behavior measured by accelerometer.

Fig 3 shows that sedentary behavior from the waist worn accelerometer did not change as a main effect of group *F*(2,65) = 1.893, *p* = 0.159), or as a main effect of time *F*(2,130) = 0.473, *p* = .473) and no main effect of time by group interaction was observed *F*(4,130) = 0.351, *p* = .843). For all three groups light intensity physical activity did not change as a main effect of group *F*(2,65) = 1.203, *p* = 0.307), or as a main effect of time *F*(2,130) = 2.568, *p* = .081), nor main effect of time by group interaction was observed *F*(4,130) = 0.897, *p* = .468). Moderate intensity physical activity did not change as a main effect of group *F*(2,65) = 2.467, *p* = 0.071), nor by time by group interaction *F*(4,130) = 1.895, *p* = .115). There was a main effect of time *F*(2,130) = 7.014, *p* = .001) with an effect size of n^2^ = .097. Overall, moderate intensity physical activity increased significantly from pre-test to week 6 (p<0.05), followed by a significant reduction from week 6 to post-test (p<0.05). No significant changes observed from pre-test to post-test (p>0.05). In general, for the three groups, vigorous intensity physical activity did not change as a main effect of group *F*(2,65) = 1.677, *p* = 0.195), it did change as a main effect of time *F*(1.416,92.018) = 4.608, *p* = .022) with an effect size of n^2^ = .066, but no main effect of time by group interaction was observed *F*(2.831, 96.384) = .272, *p* = .834).

### Movband results

**Fig 4:**
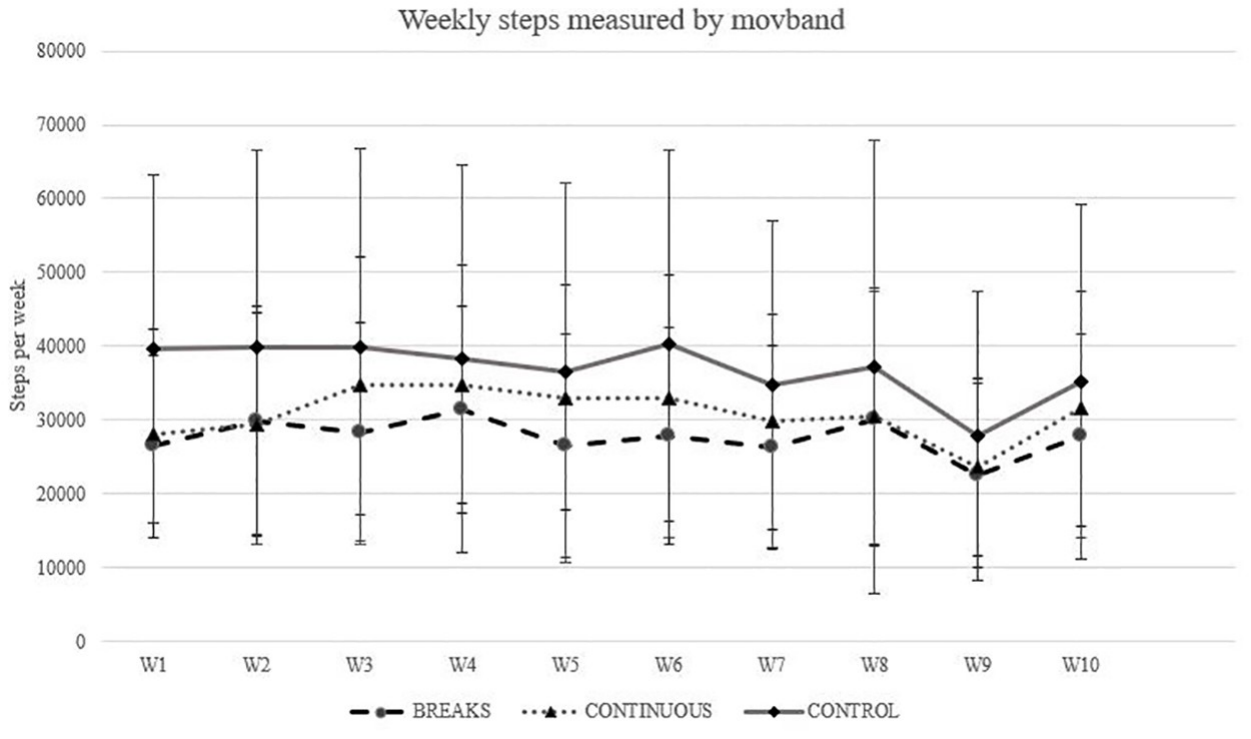
Weekly steps taken by the three groups measured with Movband.

There was a main effect of time *F*(2,130) = 3.618, *p* < .030) with a large effect size of n^2^ = .660. Overall, physical activity measured by moves increased significantly from pre-test to week 6, from pre-test to week 11, and from week 6 to week 11 (p < .001). No main effect of time by group interaction was observed *F*(4,130) = 1.021, *p* = .366) (Fig 4).

## Discussion

This study focused on a 10-week intervention for sedentary adults randomly assigned to intermittent walking, continuous walking, or control. We observed significant reductions in body weight, total fat mass, and body fat percentage in all three groups. However, aerobic fitness did not change for any group over the course of the time and physical activity measured by accelerometer indicated no significant changes in sedentary behavior or light physical activity. Moderate physical activity improved for all three groups from baseline to 6-weeks but returned to baseline measures by week 11.

In previous studies, changes in aerobic fitness have been found after a walking intervention in where intermittent and continuous walking were compared. Serwe et al. [25] found that for both models of walking, aerobic fitness, measure by 6 minutes walking tests, improved significantly. In another study, Macfarlane, et al. [46] observed significant improvements on aerobic fitness after 8-week of intervention that included continuous walking and intermittent walking groups. Karstoft et al. [47] found in a 4-month intervention that the interval-walking group increased aerobic fitness significantly after performing short bouts of walking (3x10 min/day per 8 weeks) as well as the continuous walking (30 min/day) group [48]. Different from those findings, our study did not show any significant improvement on aerobic fitness at week-6 or week-11 (Fig 2). This may be explained by the fact that in our study we instructed our participants to perform moderate intensity walking, based on a rate of perceived exertion (RPE scale). For untrained people, the self-perception of exertion may be hard to determine and actual activity does not reflect a moderate intensity [49]. Thus, it is possible that most of the participants did not achieve the physical exertion necessary to reach moderate intensity activity that would generate changes in aerobic fitness. Even though there were significant improvements on moderate intensity walking at 6-weeks, those improvements were not enough to positively affect aerobic fitness.

In our study, we observed significant positive reductions for body weight and body composition measures for all three groups. Our findings align with previous walking programs. For example, a meta-analysis [50] observed that well controlled walking programs had significant reductions on body fat. Other interventions found that intermittent walking activity produced significant reductions on fat mass [47], furthermore continuous walking interventions showed lower body fat content [51, 52]; more ambulatory physical activity accumulated and significant reductions in body fat percentages [53]. Although we did not collect dietary information, the observed changes on physical activity, at least on moderate intensity for all groups may explain changes in body composition. This is further supported by Murphy et al. [50] that showed body composition changes were associated to an incremented walking activity itself and not due to dietary changes.

Additionally, we observed similar changes in body composition for all participants, however the intermittent walking and control groups had significant improvements on lean mass and fat free mass compared to the continuous group. Karstoft et al. [47] in a well-controlled trial found that intermittent walking produced greater effects on body composition than the continuous walking, however, lean mass changes were not significant. In regards to continuous walking, Gaba et al. [54], reported that in a 10-week brisk walking intervention with women over 50 years old, no significant changes on body composition were evidenced post-intervention. For our study, breaking sedentary time through scheduled walking breaks showed positive changes in lean mass. The control group may have shown increases in lean mass due to the physical activity tracker which previous studies have demonstrated [55].

The control group showed increases in physical activity and may be due to wearing the Movband that tracks and visual displays daily moves. Past studies have found similar results and provides evidence that activity trackers are able to provide motivation or self-regulatory skills in the short term. In a preliminary study, Yuenyongchaiwat [55] found that pedometers increased physical activity and people who achieved 10k steps per day during a 12-week intervention had positive changes in body composition. In a systematic review, Bravata et al. [56] were able to determine that using pedometers, as motivators, increase physical activity and this physical activity produced significant changes in body composition. The meta-regression showed that having the pedometer and the goal of achieving 10k steps per day increased physical activity. Rooney et al. [57] gave over 500 sedentary employees with pedometers and encouraged them to walk 10k steps per day for eight weeks. They found that the pedometer was a predictor of significant improvements on physical activity. For our study, all participants were also able to see how they compared to other people within their group in terms of average daily moves. This social comparison may have provided extrinsic motivation to accumulate moves throughout the day. However, the extrinsic motivation of the wrist worn accelerometer was short lived in that the waist worn accelerometer showed no changes at week 11.

In terms of physical activity intensity, there were no physical activity differences between the two walking interventions (Fig 3). It has been theorized that short bouts of exercise may be easier for unfit people to perform, incorporate it into their schedule [17] and improve physical activity behavior. Past research has focused on the importance of eliciting moderate to vigorous physical activity to reduce risk for disease [58]. Recent and emerging literature has also shown the importance of sedentary behavior and the impact reductions in sedentary behavior has on disease risk [59] and early mortality [11]. Based on the results of this intervention, both a continuous or intermittent bout of activity are feasible as exercise prescriptions for sedentary office employees, however, an activity monitor and exercise prescription are not enough to maintain long-term walking behavior. Previous studies reported significant changes in sedentary behavior and improvements in adherence to physical activity after a walking intervention [22, 60]. However, in our study, walking activity did not translate to changes in physical activity measured by the accelerometer at post-test (Fig 3). Again, this phenomenon could be explained by the inability of our participants to accurately assess intensity levels even though they were trained on the RPE scale and experienced a “moderate pace” during baseline data collection. Based on anecdotal information provided by the participants, Thanksgiving holidays and the increase in social and work obligations contributed to the decrease in physical activity at the end of the intervention. In addition, participants reported the change to daylight savings time decreased the amount of time people could walk outside after work. This anecdotal information relates to the results obtained through the daily physical activity measured by using a wrist worn device. Data show a decrease of physical activity levels mostly seem after week 6 of the intervention (Fig 4). In support of this asseveration, a systematic review that included studies from 1980 to 2006, researchers found that during the last months of the year, people tend to do less physical activity [61]. Also, the colder weather could negatively affect physical activity levels as stated by previous studies in which researchers found a significant lower level of physical activity when cold weather is present [62, 63]. Our results support this observation, for both intervention groups, moderate intensity PA increased significantly at week six, but significantly decreased at the post-test. Based on the wrist worn accelerometer, these changes seem to occur at week 9 which corresponds to daylight savings time change. Due to the novelty of this study, more research using similar approaches is needed to explain specific variables behavior throughout a long-term intervention.

## Conclusions

In summary, when comparing the effect of intermittent vs continuous walking in our study, we could observe positive improvements from the two programs. A walking prescription for employees produced similar benefits on body weight, fat mass and body fat percentage in sedentary employees. Meanwhile, intermittent walking allowed these sedentary employees to increase lean mass and fat free mass. Intermittent walking could provide at least similar benefits on body composition compared to a continuous walking program. In addition, wearing a wrist band to track daily physical activity appears to be a short-term motivator for walking behavior, but not enough to overcome environmental barriers.

## Limitations

We consider that even though the wrist band accelerometer allowed us to observe daily physical activity, the fact that participants were able to track and see their own daily steps in the device and in the computer program after syncing the device, may have negatively affected the results of this study. Specifically, participants in the control group could be motivated by the device. For future studies we recommend blinding the device, thus, participants are not aware about steps taken per day.

Increasing evidence show that the criteria of wearing the Actigraph accelerometer 10 hours/3-5 days to assess physical activity levels may lead to underestimate physical activity achieved by participants. For example, Herrman et al. [64] suggest that the minimum accelerometer wear time should be 13 hours/day to provide valid measurement of daily physical activity. Increasing accelerometer wearing time may lead to a better estimate of sedentary behavior and physical activity and improve statistical power to write conclusions [65]. For this study wearing time shown in Table 2 indicates that all participants from the three groups worn the accelerometer above 10 hours per day which is close to what is suggested within the newer criteria.

**Table 2:** ActiGraph accelerometer average wear time ($\bar{\text{x}}\pm \text{DS}$) hours per day.

	Pre-test	Week 6	Week 11
**Intermittent group**	13.70±1.28	13.32±1.41	12.90±1.30
**Continuous group**	13.85±1.75	13.76±1.59	13.70±1.39
**Control group**	13.26±1.11	13.49±1.37	13.05±1.08

There were no significant differences in wearing time (hours per day) between groups (*F*(2,65) = 1.608, *p* = 0.208) and measurements (*F*(2,130) = 2.525, *p* = 0.084).

Another limitation we observed is the intensity at which participants walked, since they were trained to be familiar with the RPE scale to state moderate intensity while walking, it could be possible that participants were not able to reach the prescribed intensity each time they got up for a walk. Results show low percentage of time spent in this category and it may explain the lack of significant results in our variables. Untrained people may have difficulties establishing the self-perception of exertion which may lead to a different intensity level [49].

## Supporting information

S1 Dataset (xlsx) File containing data used for the analysis. (XLSX)
